# Content aware multi-focus image fusion for high-magnification blood film microscopy

**DOI:** 10.1364/BOE.448280

**Published:** 2022-01-27

**Authors:** Petru Manescu, Michael Shaw, Lydia Neary- Zajiczek, Christopher Bendkowski, Remy Claveau, Muna Elmi, Biobele J. Brown, Delmiro Fernandez-Reyes

**Affiliations:** 1 Department of Computer Science, Faculty of Engineering Sciences, University College London, London, United Kingdom; 2 Biometrology Group, National Physical Laboratory, Teddington, Middlesex, United Kingdom; 3 Department of Paediatrics, College of Medicine University of Ibadan, University College Hospital, Ibadan, Nigeria; 4 Childhood Malaria Research Group, College of Medicine University of Ibadan, University College Hospital, Ibadan, Nigeria; 5 African Computational Sciences Centre for Health and Development, University of Ibadan, Nigeria

## Abstract

Automated digital high-magnification optical microscopy is key to accelerating biology research and improving pathology clinical pathways. High magnification objectives with large numerical apertures are usually preferred to resolve the fine structural details of biological samples, but they have a very limited depth-of-field. Depending on the thickness of the sample, analysis of specimens typically requires the acquisition of multiple images at different focal planes for each field-of-view, followed by the fusion of these planes into an extended depth-of-field image. This translates into low scanning speeds, increased storage space, and processing time not suitable for high-throughput clinical use. We introduce a novel content-aware multi-focus image fusion approach based on deep learning which extends the depth-of-field of high magnification objectives effectively. We demonstrate the method with three examples, showing that highly accurate, detailed, extended depth of field images can be obtained at a lower axial sampling rate, using 2-fold fewer focal planes than normally required.

## 1. Introduction

**Fig. 1.**
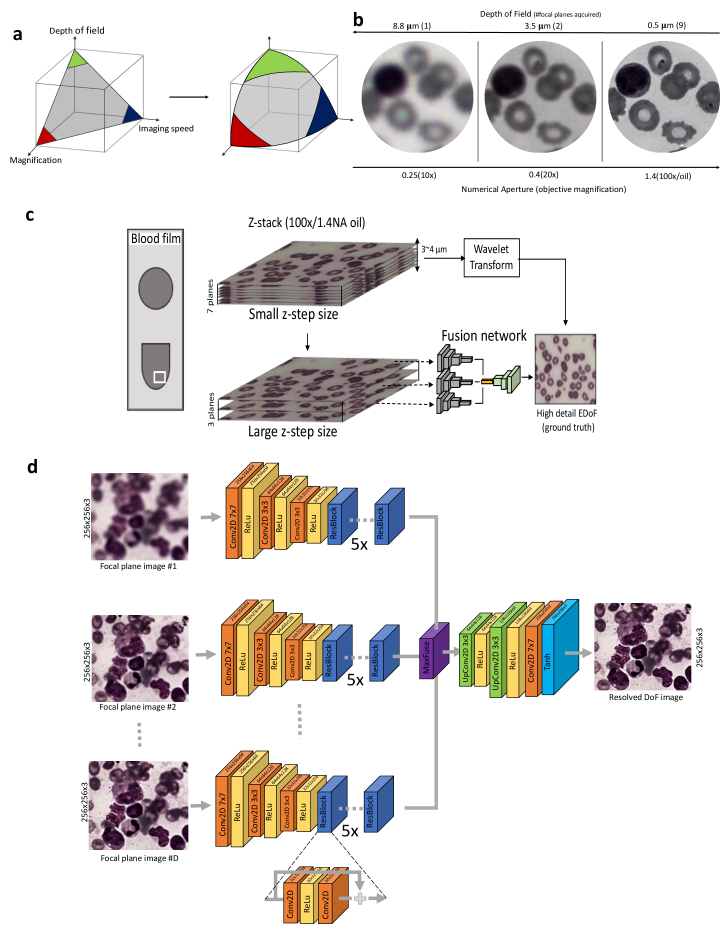
CAMI-Fusion. a) Trade-offs between imaging speed, objective magnification and depth of field in optical microscopy. CAMI-Fusion enlarges this design space. (b) Example of a blood sample imaged with low and high NA objective requiring imaging lower and higher number of focal planes respectively. The sample thickness was estimated at 4-5 µm. Malaria parasites in their ring stage can only be clearly distinguished with high NA objectives (100x) as stated in [10]. (c) Overview of the proposed pipeline for image fusion. Ground-truth EDoF generation: high resolution z-stacks with a 0.5 µm axial step size (small z-step size) are acquired using a high magnification objective (100x/1.4NA) and their corresponding extended depth of field images are computed using a wavelet-based approach (y_i_). The focal planes corresponding to a larger z-step are selected (x_i_) and passed through a convolutional neural network trained to fuse and restore y_i_ from x_i_. (d) Network architecture. CAMI-Fusion passes each focal plane through the encoder part of the network and choses the maximum activations after the residual layers before the decoder part of the network. There are 5 identical residual blocks (ResBlock) before the fusion operation. RGB Patches of 256 × 256 pixels per focal plane are used during training.

Analysis of blood specimens under a light microscope is essential to many areas of haematology from research to clinical diagnosis [1]. In contrast to methods such as flow cytometry, Coulter counters or antibody-based rapid diagnosis tests, microscopy with high magnification, high numerical aperture (NA) objective lenses allows visualization and analysis of cell morphology, indispensable in diagnosing both Communicable Diseases (CD) and Non-Communicable Diseases (NCD). The emergence of digital microscopy in haematology has recently opened the door for Artificial Intelligence (AI) assisted examination of cell morphology, in the context of blood borne parasite infections [2–4], genetic disorders [5–7] or leukaemia [8,9]. Integrated into the relevant clinical pathways, these new tools can not only alleviate the severe shortage of pathologists and expedite diagnosis but also enable precise and reproducible information retrieval from digitised specimens mitigating inherent cognitive and visual bias of the human pathologist. This requires, first of all, high-throughput digital scanning of specimens and pre-processing such that the relevant cell features in blood samples are distinguishable and can be presented to the human expert and/or to the AI assistant. Due to physical and engineering constraints, the high spatial resolution needed to analyse blood specimens comes at the expense of both the Field of View (FoV) and, more importantly, the Depth of Field: ( $\text{DoF}=\frac{\lambda }{\text{N}{\text{A}}^{2}}$ ; λ : emission wavelength, NA: numerical aperture of the objective). To capture samples thicker than the DoF, modern digital microscopes have motorized mechanical stages that move the specimens to different axial positions. This allows sequential imaging of multiple sample layers at the same *xy* position, commonly known as a z-stack. To obtain maximal axial resolution, the number of focal planes needed to be imaged ( ${\text{N}}_{\text{p}}$ ), according to Abbe diffraction limit and Nyquist sampling criterion, depends thus on both the sample thickness ( $S_{t}$ ) and the NA of the microscope ( ${\text{N}}_{\text{p}}=\frac{S_{t}\cdot {\text{NA}}^{2}}{2\lambda }$ ). In practice, the axial sampling rate is selected based on the size of the biological features of interest. For instance, a common high-resolution optical microscope with a 100x/1.4 oil immersion objective lens and a large format (2160 pixels x 2560 pixels) digital camera has a FoV of ∼140 µm x 160 µm and a DoF of ∼0.5 µm (assuming a green light). Reliable clinical assessment and diagnosis requires the analysis of a relatively large blood volume which necessitates acquisition of a large number of non-overlapping z-stacks at this magnification. As an illustration, the World Health Organization (WHO) recommends the inspection of at least 5,000 erythrocytes under such a high-magnification objective to diagnose and quantify malaria in Peripheral Blood Smears (PBS) [10]. Assuming a typical erythrocyte number density of 200 per FoV, and a sample thickness of 3-4 µ*m* this requires, in practice, imaging between 20 and 30 non-overlapping z-stacks with 7 focal planes for an axial step of ∼0.4-0.5 µ*m* [11] (for an axial resolution of ∼1 µ*m*) per z-stack (210 image acquisitions per sample). Following the same guidelines, assuming a sample thickness of 6-7 µ*m*, digital diagnosis of malaria in Thick Blood Films (TBF) requires, in practice, 100 or more non-overlapping image stacks with 10 to 14 planes [2,3] (for an axial resolution of ∼1 µ*m*) per FoV (up to 1400 image acquisitions per sample). In a similar manner, 20 to 50 FoV (up to 350 image acquisitions per sample) are inspected when diagnosing different types of leukaemia. This exemplifies that limited DoF of high-magnification objectives significantly hinders specimen imaging speed and at the same time increases data storage requirements (Fig. 1(a)).

More strikingly, an Extended DoF (EDoF) image is desired for both visual inspection and for automated AI-assisted diagnosis, which requires fusing information from z-stacks into a single plane so that all the relevant biological structures at different depths of the film are in focus. Most conventional EDoF image fusion methods are based on Multi-Scale Decompositions (MSD) [12]. They follow a three-phase structure: image decomposition, fusion and reconstruction. Such techniques, based on wavelet transforms, have been widely adopted [13,14]. However, the choice of the decomposition transform is usually made based on generic assumptions agnostic to the specific content of the z-stacks. This renders these techniques highly susceptible to noise and artefacts such as dust, fibers or air bubbles. More importantly, combining information from a relatively large number of focal plane images with such techniques becomes very computationally expensive and the methods are typically not suited for high-throughput analysis.

To find a solution to these challenges we explored the use of deep learning methods based on Convolutional Neural Networks (ConvNets) to obtain resolved EDoF images by leveraging knowledge about the image content. Deep learning methods based on ConvNets have been recently developed to achieve state-of-the-art dual-focus fusion of natural scene images [15,16]. Similar techniques have also been applied to multi-modal medical image fusion [17] or to restore in-focus images from phase coded images [18]. In low magnification microscopy, ConvNets have been employed to predict the focal position along the optical axis for Whole Slide Imaging (WSI) [19] but also to perform fast autofocusing and phase recovery in holographic imaging [20,21] or image restoration [22] in fluorescence microscopy. Others have combined wavefront encoding with ConvNets to image large areas of intact tissue without refocusing for low magnification (4x) fluorescent microscopy [23]. In wide field microscopy, a previous study showed how ConvNets can improve the reconstruction quality of full-color 3D images with an extended optical sectioning capability directly from the z-stack data acquired under a 20x objective (0.45 NA) [24]. The models trained with target images obtained with structured illumination microscopy (SIM) can significantly reduce (21-fold) the amount of raw data collection volume usually acquired with SIM [24]. None of these applications focuses on combining information from multiple focal planes (z-stacks) acquired with bright-field microscopy under high NA oil objectives (limited DoF). Here we show a solution to accelerate image acquisition at high-magnification, decrease image storage space requirements and reduce image fusion processing time by training a new content-aware multi-focus image fusion (CAMI-Fusion) ConvNet model to generate resolved high resolution EDoF images from undersampled z-stacks (Fig. 1(c)).

## 2. Methods

*Sample collection and preparation:* All biological samples were collected from participants recruited under the auspices of the CMRG at the 800-bed tertiary hospital, UCH in the city of Ibadan, Nigeria, after after de-identification of the patient information. We trained and tested the CAMI-Fusion network on three different types of samples: (a) Giemsa-stained thick blood films (TBF) and (b) Giemsa-stained peripheral blood smears (PBS) for malaria parasite detection as well as (c) Wright-stained Bone Marrow Aspirates (BMA) for diagnosing blood cancers.

*Image acquisition and pre-processing:* We used an upright brightfield microscope (BX63, Olympus, 100W halogen bulb light source) with a 100X/1.4NA oil immersion objective (MPlanApoN, Olympus) and a color digital camera (Edge 5.5C, PCO) to acquire multiple high-resolution z-stacks with an axial step of a 0.5 µm. White balancing was applied to each focal plane image. For each sample acquisition, an empty field of view (empty region of the slide not containing any blood cells) was acquired and considered as a white object reference. We computed the target high definition resolved EDoF (ground truth) for every FoV stack applying a wavelet decomposition-based method [13] to the full z-stack. Undersampled z-stacks (network input) were obtained by increasing the axial step size from 0.5 µm to 1 µm and 2 µm.

*Network architecture and training:* CAMI-Fusion takes as input a stack of focal plane images, passes each of them through a series of two-dimensional convolutional layers, fuses the resulting high-dimensional transformed focal plane images into one high dimensional feature tensor and then reverts back to the image space through a series transposed two-dimensional convolutional layers (Fig. 1(c)). CAMI-Fusion relies on a a residual ConvNet multiple input encoder-decoder architecture which can be formulated mathematically as Eq. 1:

(1) $$\begin{array}{c} O=\tanh(C_{2D}(max({ℜ}_{2D}(E_{2D}(I_{1})),\ldots \;{ℜ}_{2D}(E_{2D}(I_{D}))))) \end{array}$$

where $I_{1},\ldots I_{D}$ are the *D* focal planes acquired at different depth (size WxHx3), and max() is element-wise maximum or max-pooling applied to the output tensors ${ℜ}_{2D}(E_{2D}(I_{i}))$ . $E_{2D}$ represents the encoder (output size: $\frac{W}{2^{N_{L}}}\;\text{x}\frac{H}{2^{N_{L}}}\text{x}E_{d}$ ), ${ℜ}_{2D}$ the residual layers (output size: $\frac{W}{2^{N_{L}}}\;\text{x}\frac{H}{2^{N_{L}}}\text{x}E_{d}$ ), $C_{2D}$ the decoder part of the network (output size: WxHx3) and *O* the output color image with:

$$\{\begin{array}{c} W,\;H:width,\;height\\ E_{d}:encoder\;depth\\ N_{L}:number\;of\;convolutional\;layers\\ D:depth,\;or\;number\;of\;z-stacks \end{array}$$

The loss function consisted of three components: the pixel-wise mean absolute error $l_{MAE}$ , the structural similarity index $l_{SSIM}$ and pixel-wise mean absolute error of the Fourier Transform $l_{FFT}$ of the target image and the network output image, which can be formulated mathematically as Eq. 2:

(2) $$\begin{array}{c} l=\lambda_{1}l_{MAE}+\lambda_{2}l_{SSIM}\;+\lambda_{3}l_{FFT}\; \end{array}$$

with $\lambda_{1},\;\lambda_{2},\;\lambda_{3}$ hyper-parameters controlling the relative weight of each loss component. Network models were trained using patches of 256 × 256 RGB pixels randomly cropped from acquired stacks (Fig. 1(c)). The loss weights were set at $\lambda_{1}$ =1.0, $\lambda_{2}=$ 0.1, $\lambda_{3}$ =0.5. A batch size of 2 and an Adam optimizer with a learning rate of 1e-4 were employed during training. The proposed architecture was implemented in Python using Tensorflow.

## 3. Results

### 3.1 CAMI-Fusion efficiently generates EDoF images of thick-blood-films suitable for AI-assisted malaria diagnosis

**Fig. 2.**
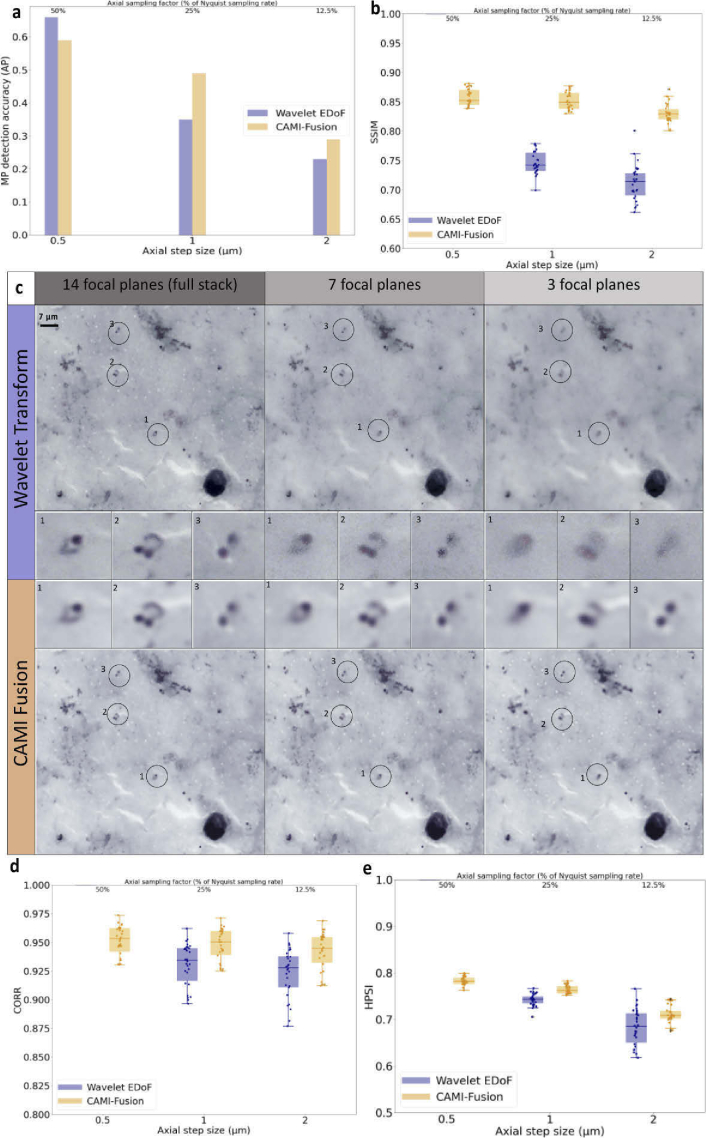
Results on TBF malaria samples. (a) Malaria Parasite (MP) detection accuracy in terms of Average Precision (AP) of a RetinaNet object detector trained to detect MP in high resolution EDoF image fields obtained by fusing 14 focal planes. RetinaNet was tested on 30 different image fields obtained by fusing 14, 7 and 3 planes respectively using the wavelet transform approach and CAMI-Fusion. (b) Image quality assessment for the three different axial steps values. Box-dot plots (n = 30) show SSIM (higher is better) for the resolved EDoF images obtained with wavelet transform fusion approach and the images obtained with CAMI-Fusion. (c)Image fusion outputs for thick blood film stained with Giemsa used in malaria diagnosis. Shown are fusion results using the wavelet transform approach and CAMI-fusion for 14, 7 and 3 focal planes respectively. Malaria parasites are highlighted. (d-e) Additional image similarity comparisons. CORR: Pearson Correlation Coefficient [26]. HPSI: Haar wavelet-based perceptual similarity index [27]. Comparison of the results obtained using combined loss function during training the fusion models with a simple loss function (L1) can be found in Fig. S1 (See Supplemental document 1).

*Plasmodium falciparum* malaria remains one of the greatest world health burdens with over 200 million cases globally leading to half-million deaths annually, mostly among children under five years of age [25]. Currently, the gold standard for malaria diagnosis is the microscopic evaluation of Giemsa stained thick blood smears by trained malaria pathologists [25]. Due to the small size of the ring-stage parasites in circulation (∼ 2 to 3 µm) high magnification oil-objectives (100x) with a large NA (typically 1.4) are recommended to distinguish the parasites from distractors. Recently, computer vision techniques have attempted to automatically detect malaria parasites in digitized EDoF images of thick blood smears [2–4]. Here, we acquired multiple FoV, each comprising 14 focal planes separated by 0.5 µm, from TBF samples showing different levels of parasitemia, ensuring a total depth of 6.5 µm for each FoV. The MSD wavelet-based resolved EDoF [13] method was applied to the 14 focal planes to produce a target (ground truth) high resolution EDoF resolved image corresponding to each z-stack. CAMI Fusion was trained to generate these target images by fusing undersampled z-stacks acquired using an axial sampling rate of 1 µm (7 focal planes) and 2 µm (3 focal planes). The CAMI-Fusion ConvNet model was trained using 159 z-stacks and tested on 30 z-stacks, each covering an area of 166 µm x 142 µm. We observed a loss of structural details using the wavelet based resolved EDoF test images as the axial sampling rate decreased from 0.5 µm (14 planes) to 1 µm (7 planes) and 2 µm (3 planes) (Fig. 2(c)). In contrast, the resolved EDoF images obtained with the CAMI Fusion model maintained almost the same level of detail as the ground truth high resolution images (Fig. 2(c)). To quantify this observation, we first measured the similarity between prediction and ground-truth images for three different z-step sizes.

The structural similarity index (SSIM) [28] improved considerably (by more than 14%) compared against results obtained with the wavelet transform extended EDoF method as the axial sampling rate decreased from 0.5 µm (14 planes) to 1 µm (7 planes) and 2 µm (3 planes) (Fig. 2(b)). We next asked whether CAMI fusion is compatible with common automatic analysis tasks in TBF microscopy, such as malaria parasite detection. To test this we measured the performance of a RetinaNet object detector [29] model trained to detect malaria parasites (MPs) [2] on test images generated using the wavelet transform method and the CAMI fusion for the three different axial step sizes (Fig. 2(a)). The MP detection accuracy in terms of average precision (AP; area under the precision-recall curve) improved from AP = 0.35 (0.23) on the wavelet transform based resolved DoF test images to AP = 0.49 (0.29) on the same test resolved DoF images obtained with the network model for an axial step size of 1 µ*m* (2 µ*m*).

### 3.2 CAMI-Fusion efficiently generates EDoF of peripheral blood samples and bone marrow aspirates thin-blood-films

We further investigated whether CAMI-Fusion preserves details of erythrocytes and leukocytes when capturing undersampled z-stacks from peripheral blood smears (PBS) and bone marrow aspirates (BMA) with an axial step double the size than used in clinical and research settings. The specific regions of PBS and BMA, containing spatially separated blood cells useful for morphological and textural analysis are ∼3-4 µm thick. First, we used the same 100x/1.4NA oil objective to acquire multiple z-stacks of 7 planes each with an axial step of 0.5 µm from PBS Giemsa stained samples. In a similar setup as the TBF application, CAMI-Fusion was trained to generate resolved EDoF images using an axial step of 1 µm on 64 z-stacks and tested on 13 separate z-stacks.

**Fig. 3.**
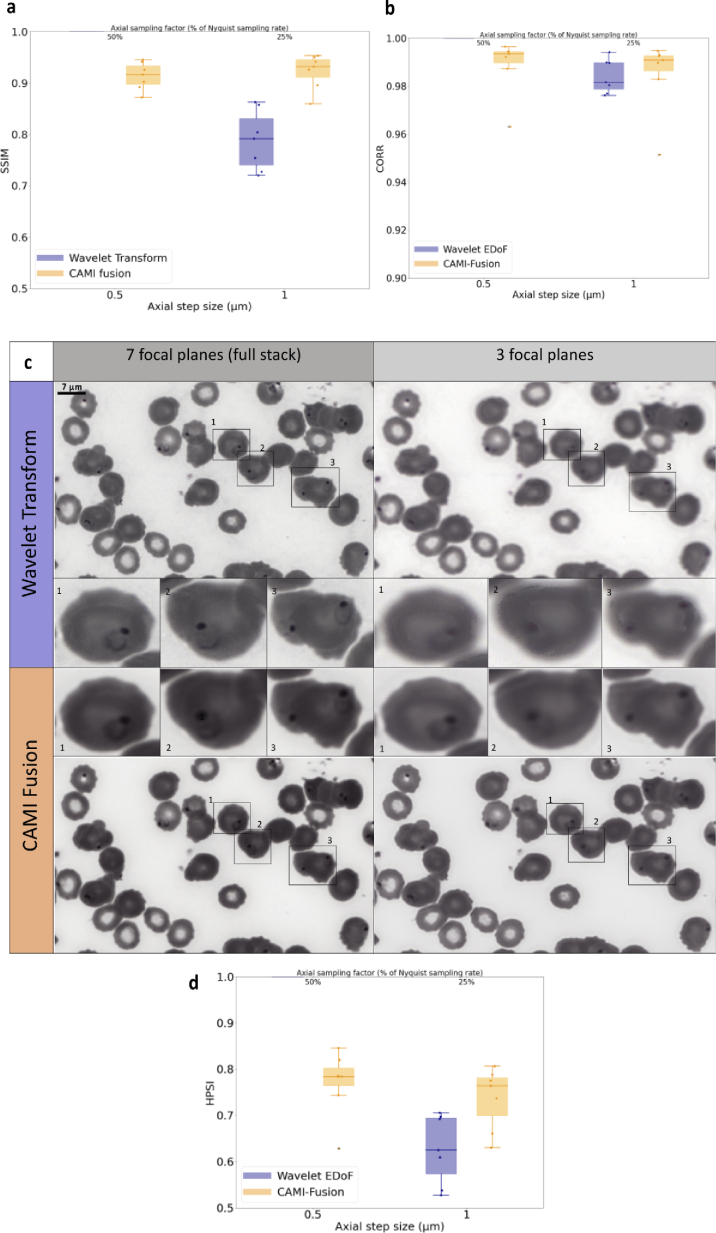
Fusion of image stacks from peripheral blood smears. (a) Image quality assessment for 1 µm axial step values compared to 0.5 um. Box-dot plots (n = 14) show SSIM (higher is better) for the resolved EDoF images obtained with wavelet transform fusion approach and the images obtained with CAMI-Fusion. (c) Image fusion outputs for PBS stained with Giemsa used in malaria diagnosis. Shown are fusion results using the wavelet transform approach and CAMI-fusion for 7 (0.5 µm axial step size) and 3 (1 µm axial step size) focal planes respectively. Red blood cells infected with malaria parasites are highlighted. (b)-(d) Additional image similarity comparisons. CORR: Pearson Correlation Coefficient [26]. HPSI: Haar wavelet-based perceptual similarity index [27].

**Fig. 4.**
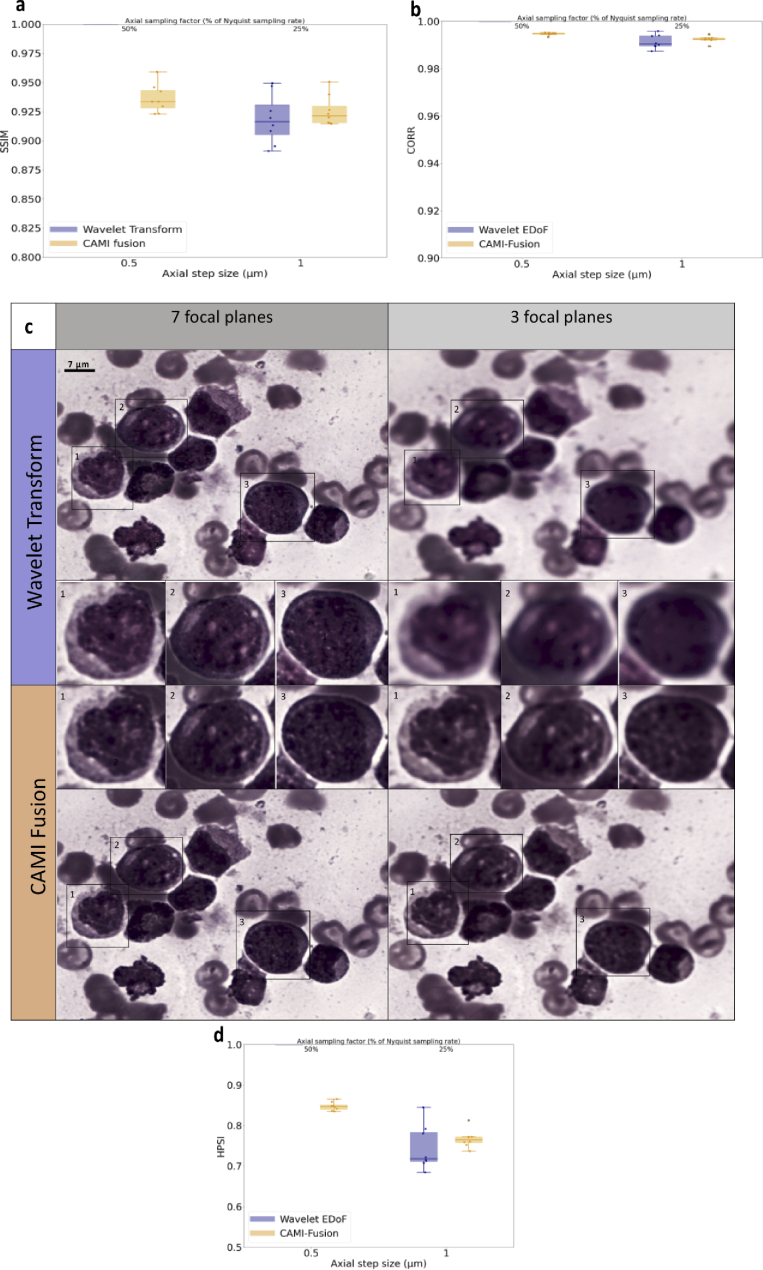
Fusion of image stacks from bone marrow aspirates. (a) Image quality assessment for 1 µm axial step values compared to 0.5 um. Box-dot plots (n = 8) show SSIM (higher is better) for the resolved EDoF images obtained with wavelet transform fusion approach and the images obtained with CAMI-Fusion. (c) Image fusion outputs for BMA stained with Wright used in AML diagnosis. Shown are fusion results using the wavelet transform approach and CAMI-fusion for 7 and 3 focal planes respectively. White blood cells are highlighted. (b)-(d) Additional image similarity comparisons. CORR: Pearson Correlation Coefficient [26]. HPSI: Haar wavelet-based perceptual similarity index [27].

We observed that, in contrast to the wavelet-based fusion, the resolved EDoF images output by the network preserved both cell sharpness and MP ring shape (Fig. 3(c)) when using only half of the focal plane images (corresponding to an axial step of 1 µm). The SSIM values between the full-stack target resolved DoF image and the DoF images generated using half the focal planes values enforce this observation (Fig. 3(b)). The same setup was employed to image Wright-stained BMA from samples presenting acute myeloid leukemia (AML) and non-Hodgkin lymphoma (NHL). In an identical manner to the PBS application, CAMI-Fusion was trained to generate resolved EDoF images of BMA with a z-step of 1 µm on 42 z-stacks and tested on 9 separate z-stacks (Fig. 4). Contrary to the classical wavelet transform based fused images, the output of the network preserves both image contrast and the fine structures of the white blood cell nuclei essential for blood cancer diagnosis (Fig. 4(c)).

### 3.3 CAMI-Fusion speeds up data acquisition and processing of blood films

**Fig. 5.**
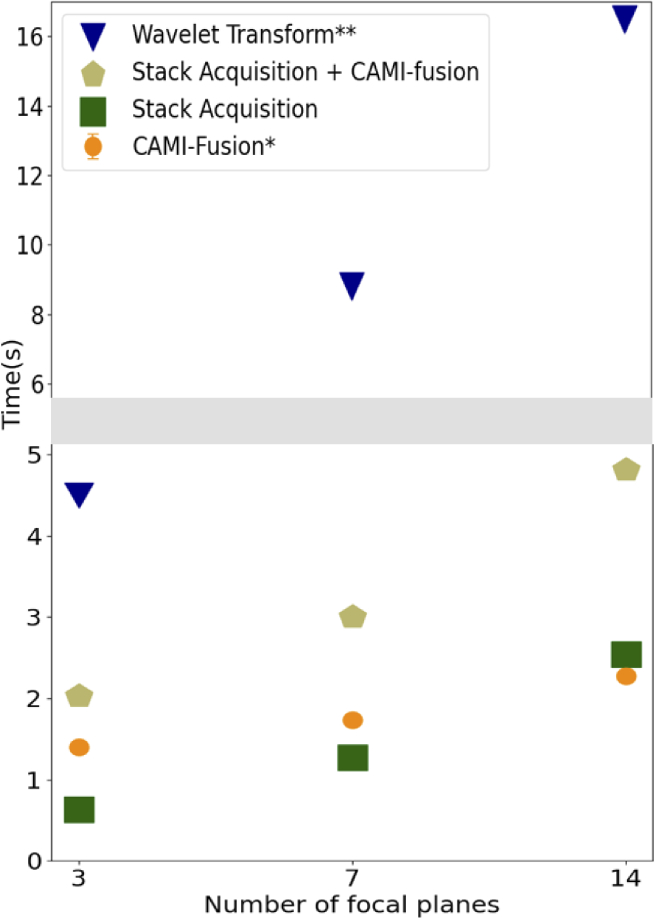
Z-stack acquisition and processing times. The processing times were measured on an Intel Core i9 3.1 GHZ CPU with a NVIDIA GeForce RTX GPU with 12 Gb of memory. *Cami-fusion makes use of GPU capabilities. **The Wavelet-based EDoF [13] was implemented in Matlab and does not use GPU capabilities.

Figure 5 shows an example of measured imaging and processing speed as a function of the number of focal planes. Under the current conditions, using 7 focal planes instead of 14 leads up to a 40% economy in the overall time budget (image acquisition and fusion) while going from 7 to 3 decreases the overall time by approximately 33%. CAMI-fusion takes advantage of the fast GPU implementation and reduced the computational time by up to a factor of 8 compared to the CPU wavelet-based implementation.

## 4. Discussion

We introduced CAMI-Fusion networks designed to perform multi-focus image fusion of high-magnification bright field microscopy z-stacks with larger axial steps than the theoretical depth-of-focus of the objective. Experiments showed that clinically relevant details can be preserved when combining half or less focal planes than theoretically needed. This can potentially speed up both the image capture of blood specimens and the fusion process by a factor of two, effectively increasing the sample acquisition and analysis rate. A key feature of our approach is that CAMI-Fusion implicitly learns how to perform both image fusion and image restoration at the same time. This not only allows imaging using fewer focal planes to obtain an accurate result, but also increases robustness to axial steps allowing the potential use of cheaper hardware which can lead to better scaling of digital high magnification microscopy, especially in resource constrained settings. Another potential advantage of learning to fuse z-stacks based on their content rather than using generic decompositions is the robustness to artefacts such as dust, air bubbles or staining accidents. In theory, CAMI-Fusion would be able to learn how to remove such artefacts from the resolved EDoF images. However, to generalize such an artefact robust fusion model and apply it in clinical settings, the CAMI-Fusion networks need more training samples verified by human microscopists. We have trained three distinct models for three applications. By increasing the training samples, a unique more generic model be able to learn how to fuse different types of images. Stain normalisation techniques [30] can be employed to render CAMI-fusion models robust to the variations in the staining procedure and imaging conditions present in different centres.

More generally the CAMI-Fusion method has potential to be extended to other bioimaging applications such as high-resolution histopathological imaging of tissue sections, where it can accelerate WSI of relatively thick samples. Live imaging of cells and model organisms is another area which would benefit from a CAMI-Fusion approach. By enabling the recovery of accurate structural images from undersampled focal series, the method offers the possibility of increasing image acquisition speed to better capture dynamic events and, perhaps more importantly, reducing overall light exposure for sensitive live biological samples to limit adverse phototoxic effects.

## Data Availability

Data underlying the results presented in this paper are available in Ref. [31].

## Supplemental document

See Supplement 1 for supporting content.
