# Supplementary material for: Content aware multi-focus image fusion for high-magnification blood film microscopy
Source: Biomed Opt Express. 2022 Jan 27;13(2):1005–16. doi: 10.1364/BOE.448280 (PMC8884220; doi:10.1364/BOE.448280)
Supplement: Supplementary file 1 [file boe-13-2-1005-s001.pdf]

Supplemental Document

Biomedical Optics EXPRESS

Content aware multi-focus image fusion for high-magnification blood film microscopy: supplement

PETRU MANESCU,[1] MICHAEL SHAW,[1,2] LYDIA NEARY- ZAJICZEK,[1] CHRISTOPHER BENDKOWSKI,[1] REMY CLAVEAU,[1] MUNA ELMI,[1] BIOBELE J. BROWN,[3,4,5] AND DELMIRO FERNANDEZ-REYES[1,3,4,5,*]

[1]*Department of Computer Science, Faculty of Engineering Sciences, University College London, London, United Kingdom*
[2]*Biometrology Group, National Physical Laboratory, Teddington, Middlesex, United Kingdom*
[3]*Department of Paediatrics, College of Medicine University of Ibadan, University College Hospital, Ibadan, Nigeria*
[4]*Childhood Malaria Research Group, College of Medicine University of Ibadan, University College Hospital, Ibadan, Nigeria*
[5]*African Computational Sciences Centre for Health and Development, University of Ibadan, Nigeria*
[*]*Delmiro.Fernandez-Reyes@ucl.ac.uk*

This supplement published with Optica Publishing Group on 27 January 2022 by The Authors under the terms of the Creative Commons Attribution 4.0 License in the format provided by the authors and unedited. Further distribution of this work must maintain attribution to the author(s) and the published article's title, journal citation, and DOI.

Supplement DOI: https://doi.org/10.6084/m9.figshare.17912330

Parent Article DOI: https://doi.org/10.1364/BOE.448280

Content Aware Multi-focus Image Fusion for High-Magnification Blood Film Microscopy : supplemental document

The use of the combined loss proposed in equation (2) during training improves the fusion outputs compared to a simple L1 (MAE) loss (Fig. S1).

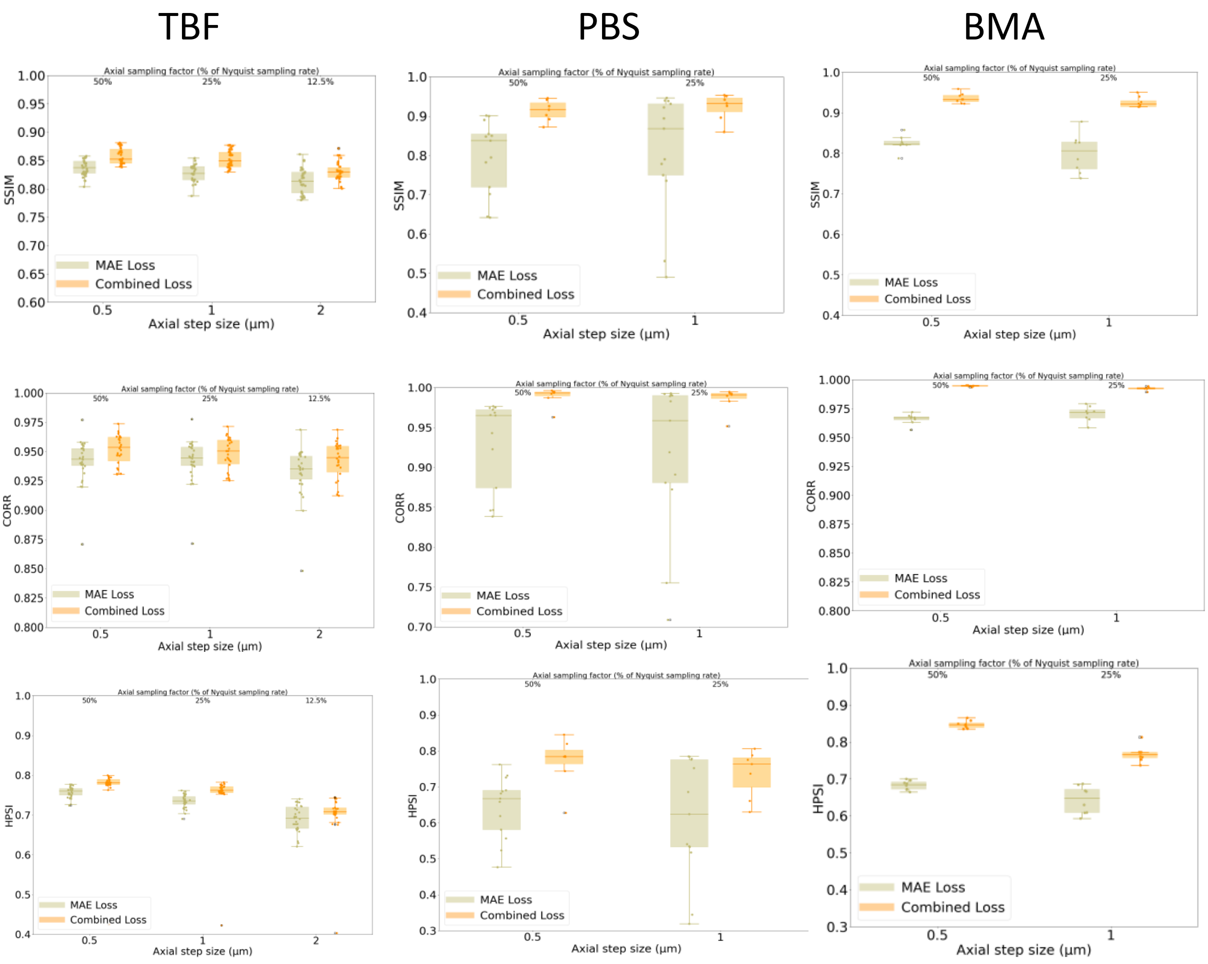

Fig. S1. Effect of the combined loss function during training on CAMI fusion models applied to the test dataset with respect to the ground-truth EDoF obtained with the full stacks (axial step size 0.5). TBF: Thick Blood Film. PBS: Peripheral Blood Smear. BMA: Bone Marrow Aspirate. SSIM: Structural Similarity Index [25]. CORR: Pearson Correlation Coefficient [27]. HPSI: Haar wavelet-based perceptual similarity index [28].
